# The effects of water-pipe smoking on birth weight: a population-based prospective cohort study in southern Iran

**DOI:** 10.4178/epih.e2018008

**Published:** 2018-03-13

**Authors:** Shahrzad Nematollahi, Mohammad Ali Mansournia, Abbas Rahimi Foroushani, Mahmood Mahmoodi, Azin Alavi, Mohammad Shekari, Kourosh Holakouie-Naieni

**Affiliations:** 1 Department of Epidemiology and Biostatistics, School of Public Health, Tehran University of Medical Sciences, Tehran, Iran; 2 Department of Gynecology and Obstetrics, School of Medicine, Hormozgan University of Medical Sciences, Bandar Abbas, Iran; 3 Department of Medical Genetics, School of Medicine, Hormozgan University of Medical Sciences, Bandar Abbas, Iran; 4 Bandar Abbas Health Research Station, World Health Organization Regional Malaria Training Center, Tehran University of Medical Sciences, Tehran, Iran

**Keywords:** Low birth weight, Water-pipe smoking, Prospective studies, Suburban population, Iran

## Abstract

**OBJECTIVES:**

Consecutive community health assessments revealed that water-pipe smoking in women and impaired growth in children were among the main health concerns in suburban communities in southern Iran. The aim of the present study was to identify the effects of water-pipe smoking during pregnancy on birth weight.

**METHODS:**

Data from a population-based prospective cohort study of 714 singleton live pregnancies in the suburbs of Bandar Abbas in southern Iran in 2016-2018 were used in this study. Data about water-pipe smoking patterns and birth weight were collected by questionnaires during and after the pregnancy. Low birth weight (LBW) was defined as a birth weight below 2,500 g. Statistical analyses were performed using generalized linear models, and the results were presented in terms of relative risk (RR) and 95% confidence intervals (CI).

**RESULTS:**

Fifty (8.2%) of the study subjects smoked water-pipe. The adjusted risk of LBW increased 2-fold in water-pipe smokers (adjusted RR [aRR], 2.09; 95% CI, 1.18 to 3.71), and by 2.0% for each 1-year increase in the duration of water-pipe smoking (aRR, 1.02; 95% CI, 0.99 to 1.05).

**CONCLUSIONS:**

Our results showed that water-pipe smoking during pregnancy was an important risk factor for LBW in this population sample from southern Iran. The introduction of regulations onto prevent water-pipe smoking and the implementation of community health action plans aiming at empowering women and increasing women’s knowledge and awareness regarding the health consequences of water-pipe smoking are proposed.

## INTRODUCTION

According to the results of 2 consecutive community health assessments in the suburban areas of Bandar Abbas, the capital of Hormozgan Province in southern Iran [1], impaired fetal and child growth and the high prevalence of water-pipe smoking in women were persistent health concerns of the community members [2]. Low birth weight (LBW), defined as a birth weight below 2,500 g, is a universal indicator of health status and is associated with an elevated risk of mortality and morbidity [3]. The prevalence of LBW ranges from 5.0 to 8.0% in Iran, and it has been associated with lower socioeconomic status (SES) [4-6]. LBW, which results from inadequate fetal weight gain in utero, is influenced by many interrelated risk factors, such as genetics, environmental exposures, and maternal characteristics and lifestyle, especially tobacco smoking [7,8]. Due to the well-established adverse effects of cigarette smoking on pregnancy outcomes [9], attention has been paid to other types of tobacco use, such as water-pipe smoking. The tradition of water-pipe smoking (also known as narghile, hookah, and shisha) dates back 400 years in Turkey, India, and Iran [10], and recent reports have shown that 8.0-14.0% of pregnant women in southern Iran use water-pipe regularly [11,12]. The adverse effect of water-pipe smoking on birth weight has been the focus of very limited research, including a study showing that the odds of intrauterine growth retardation increased by 3.5 times in water-pipe smoking mothers in Bandar Abbas [13]. Moreover, a meta-analysis of the effects of water-pipe on pregnancy outcomes showed an odds ratio (OR) of 2.39 for LBW [14]. Despite the high prevalence of water-pipe smoking in women in Middle Eastern countries (6.0-34.0%), there is limited valid epidemiologic evidence regarding its health consequences in Iranian women. Therefore, the objective of the present study was to identify the magnitude of the effect of water-pipe smoking on birth weight in a prospective study of pregnant women in suburban communities in southern Iran.

## MATERIALS AND METHODS

This study used data from the first and second phases of a prospective cohort study entitled “A population-based prospective cohort study to identify contributors of mother and child health in suburban communities” in Bandar Abbas, the capital of Hormozgan Province, with an estimated population of 680,366 in 2016. According to the Human Development Index (HDI) report for 2013, Hormozgan Province was categorized as an unindustrialized region, with a rank of 17 among the 31 provinces in Iran and an HDI of 0.704 [15].

The project was designed to follow-up 1,000 pregnant women recruited through a home-by-home inquiry. The source population consisted of all pregnant women residing in the 3 most socially and economically vulnerable neighborhoods of Bandar Abbas. Four structured questionnaires and laboratory records were used to collect data from the mothers and their children at 4 visits, ranging from pregnancy to 1, 6, and 12 months after birth. The main objectives were to monitor the children’s growth and to investigate the effects of lifestyle-related and environmental factors of the mothers and their families on the children’s health. The study protocol was approved by the institutional review board of the National Institute for Medical Research Development (NIMAD) (approval code: 943607 and ethical code: N. IR.NIMAD.REC.1396. 205). The details of the methodology have been published elsewhere [2]. At the time of the present study (September 2017), data from 714 pregnant women were available (response rate, 99.0%). Sixty-nine (10.0%) pregnancies ended in miscarriage (i.e., spontaneous termination of the pregnancy before 24 weeks of gestation), and 38 (5.3%) ended in preterm birth (i.e., birth before 37 weeks of gestation) [16]; therefore, the sample size was restricted to 607 pregnancies with full-term singleton live births. Due to the small number of cigarette smokers in our sample (n= 2), they were excluded from the analysis, and hence the final analysis included 605 women. For the purposes of the present study, the explanatory variables collected from the interviews included maternal demographics (age, education, and occupation), pattern of prenatal visits, dietary supplementation intake, and obstetrics history (gestational age, birth order, gravidity and parity, and history of miscarriage or LBW). The SES of the household was measured based on the number of rooms, family size, and ownership of 9 assets (private car/motorcycle, refrigerator, freezer, washing machine, dishwasher, vacuum cleaner, LED television, and personal computer/laptop). Our main outcome was LBW, defined as a birth weight below 2,500 g. The main exposure variable was water-pipe smoking. The subjects were asked if they ever smoked water-pipe, and data on the age at starting water-pipe smoking, the duration of water-pipe smoking, smoking water-pipe during the current pregnancy, number of water-pipe sessions per day, and second-hand water-pipe smoking (exposure to water-pipe smoke at home or work) were also gathered. The main exposures were water-pipe smoking during the current pregnancy (yes/no), number of water pipe sessions per day, age at starting water-pipe smoking (in years), and duration of water-pipe smoking (in years). The dependent variable was the weight of the newborn, which was classified as equal to or below 2,500 g (LBW), or as above 2,500 g (normal). The SES of the household was derived using principal component analysis [17], and the first 3 components, including household assets, monthly expenditures, and household size (floor area per capita), were selected. These 3 variables explained 49.0% of the total variance.

Bivariate comparisons were performed using the chi-square test. Confounder selection was based on the change-in-estimate strategy. Accordingly, confounders were selected if the change in exposure effect estimator adjusted for the covariates fell outside the equivalence range of 0.9 to 1.1 for the relative risks (RRs) [18]. Hence, the potential confounders were gestational age, monthly expenditures, household size, household assets, regular prenatal visits, and second-hand water-pipe smoking. The adjusted effects of the exposure were estimated using generalized linear models in the form of log-binomial models with a logarithmic link function to estimate the RRs of LBW with 95% confidence intervals (CIs). Data were analyzed using Stata version 13 (StataCorp., College Station, TX, USA). The p-values< 0.05 were considered to indicate statistical significance.

## RESULTS

Fifty (8.2%) pregnant women smoked water-pipe, with an average of 1.37 water-pipe sessions per day. The mean± standard deviation (SD) age at starting water-pipe smoking was 18.24± 0.66 years, while 14 (28.0%) started smoking before the age of 15 years. The mean duration of water-pipe smoking was 9.10± 0.91 years, and most of the smokers (62.0%) only engaged in 1 water-pipe session per day. Water-pipe smoking was reported more often among unemployed pregnant women, those living in high-density families, and those in households with monthly expenditures of less than USD 160. It was also more common among women with no antenatal care and no intake of fresh fruits or vegetables.

Overall, 155 (25.6%) pregnant women were second-hand water-pipe smokers; with an average of 1.86± 0.06 hr/d and 2.10± 0.06 d/wk of exposure to water-pipe smoke (Table 1).

The risk of LBW was estimated to be 11.7% (n= 71), and was significantly higher in water-pipe smokers (24.0 vs. 10.6% in non-smokers, p= 0.005). Mothers who started water-pipe smoking before the age of 15 years and those who had engaged in water-pipe smoking for more than 10 years had the highest proportions of LBW infants (47.6 and 37.0%, respectively) (Table 2).

The final model showed that the adjusted risk of LBW increased significantly by 2-fold in water-pipe smokers (adjusted RR [aRR], 2.09; 95% CI, 1.18 to 3.71). Compared to non-smokers, the risk of LBW increased by 2.0% with each 1-year increase in the duration of water-pipe smoking (aRR, 1.02; 95% CI, 0.99 to 1.05) (Table 3).

## DISCUSSION

Although it has spread throughout the world, water-pipe smoking is still most pronounced in the eastern Mediterranean region [19]. We found that 8.2% of pregnant women in our study in southern Iran smoked water-pipe, which is quite similar to the results of studies conducted in Lebanon and Jordan (4-9%) [20,21]. In accordance with previous studies [21,22], most water-pipe smokers in our sample had low SES, more irregular prenatal visits, and more exposure to water-pipe smoke.

Overall, 11.7% of the newborns in our sample were LBW. After adjusting for confounders, we found that smoking water-pipe during pregnancy increased the risk of LBW by 2-fold. In a study in Lebanon, Rachidi et al. [20] reported that compared to non-smoking mothers, mothers who smoked either water-pipe or cigarettes had 79% greater odds of having an underweight infant. However, they did not differentiate between the effects of cigarettes and those of water-pipe. In a study in Bandar Abbas, Aghamolaei et al. [13] found 3.5 times higher odds of intrauterine growth retardation in water-pipe smokers. Tamim et al. [10] showed that smoking water-pipe during pregnancy could increase the risk of LBW by 2.4 times, although the mean birth weight remained statistically unchanged. Furthermore, a meta-analysis estimated a pooled OR of 2.39 for the effect of water-pipe smoking in pregnancy on LBW [23].

The mechanism by which water-pipe smoking affects fetal growth is not fully understood. Nevertheless, compared to cigarette smoking, exclusive water-pipe smoking tends to produce higher concentrations of respirable particulate matter such as carbon monoxide, polycyclic aromatic hydrocarbons, and volatile aldehydes. Compared to a cigarette, a single session of water-pipe smoking, which usually takes 45 minutes, generates more than 40 times the smoke volume [24]. Water-pipe smoking creates as much nicotine and carboxyhemoglobin as cigarette smoking [24], which may be responsible for impaired fetal development. The concentration of nicotine in the placenta is 15% higher than its concentration in the maternal blood. By inducing maternal catecholamine release, nicotine causes uterine vasoconstriction, crosses the placenta, and targets specific neurotransmitter receptors in the fetal brain, thereby increasing susceptibility to hypoxia-induced brain damage [25]. Furthermore, maternal smoking increases carboxyhemoglobin levels in the umbilical arteries, resulting in fetal hypoxia [26]. The relationship between the number of daily water-pipe sessions and fetal growth has been investigated to some extent in previous studies. Nuwayhid et al. [27], for instance, showed that having more water-pipe sessions per day could reduce the mean birth weight by 100 g. Tamim et al. [10] also showed that more water-pipe sessions per day increased the risk of LBW, with an unchanged mean birth weight. Nonetheless, the evidence regarding the effect of the number of water-pipe sessions on fetal growth is still scarce.

Simultaneously with the growing trend of water-pipe smoking worldwide, second-hand smoking has also become a global public health challenge. Smoking water-pipe has such a strong social dimension that many smokers practice it in the company of friends and family members [19]. Exposure to water-pipe smoke causes a higher blood concentration of toxins, metals (e.g., lead), carbon monoxide, tar, nicotine, polycyclic aromatic hydrocarbons and aldehydes, all of which are detrimental to the health of the fetus [28]. We found that one-quarter of the non-water-pipe smokers in our sample were second-hand water-pipe smokers, and the proportion of LBW infants was significantly higher in this group. This finding is consistent with previous reports on the adverse effects of passive water-pipe smoking on birth weight [21,29].

The recent global epidemic of water-pipe is attributed to various factors such as social acceptability and the lack of water-pipe specific regulations [30]. Although the Islamic Republic of Iran received the highest score in the World Health Organization MPOWER tobacco control program among countries in the Eastern Mediterranean Regional Office, the enforcement has focused on cigarette smoking [31], while measures such as monitoring water-pipe use and raising taxation on water-pipe are still in progress.

Moreover, warning about the hazards of tobacco is worth further attention as an aspect of the global tobacco control strategy. People’s positive attitudes and perceptions towards water-pipe are partially responsible for its spread [32]. Similarly, the results of our previous work confirmed that women had favorable attitudes regarding water-pipe smoking in Bandar Abbas (data unpublished). Accordingly, community health action plans with the aim of increasing women’s knowledge and awareness regarding the health consequences of water-pipe smoking are highly warranted. Such action plans, based on the results of our prospective cohort in the area, are currently under development by the investigators of the present study. Targeting women and their families in the most socially and economically vulnerable population groups, the action plans aim at educating and empowering women to quit water-pipe smoking and to avoid water-pipe smoke. Community health action plans, as the final phase of the community health assessment process, require collaboration of the community members and local stakeholders for them to be implemented [1].

This study was among the first attempts to identify the effects of water-pipe smoking during pregnancy on neonatal health in Iran. The prospective nature of the study removed biases that could have stemmed from retrospective data collection, and the home-by-home sampling strategy ensured generalizable and externally valid results.

However, our study suffered from some limitations. First, using interviews as the main data collection strategy probably underestimated the prevalence of water-pipe smoking and second-hand exposure [33]. Second, comparing the effects of water-pipe smoking across studies can be challenging because the amount of tobacco inhaled during a single water-pipe session is highly dependent on details of the apparatus used, including the set-up of the tubes and the water. The lack of a standard dose for a single waterpipe smoking session makes investigating the effects of water-pipe cumbersome.

In conclusion, our results showed that water-pipe smoking during pregnancy was an important risk factor for LBW in this population sample from southern Iran. The introduction of regulations on water-pipe smoking and the implementation of community health action plans aiming at empowering women and increasing women’s knowledge and awareness regarding the health consequences of water-pipe smoking are proposed.

## Figures and Tables

**Table 1.** General characteristics of pregnant women according to water-pipe smoking status in Bandar Abbas, Iran, 2016-2017

Variable	Total	Water-pipe smoking		p-value
		No	Yes	
Maternal age (yr)				0.81
<20	37 (6.1)	34 (91.8)	3 (8.1)	
20-35	490 (80.9)	448 (91.4)	42 (8.5)	
>35	78 (12.8)	73 (93.5)	5 (6.4)	
Education level				0.11
Illiterate	20 (3.3)	19 (95.0)	1 (5.0)	
Reading and writing	151 (24.9)	132 (87.4)	19 (12.5)	
High-school or diploma	344 (56.8)	318 (92.4)	26 (7.5)	
College or higher	90 (14.8)	86 (95.5)	4 (4.4)	
Occupation				0.72
Unemployed	588 (97.1)	539 (91.6)	49 (8.3)	
Employed	17 (2.8)	16 (94.1)	1 (5.8)	
Household size				0.45
<4	345 (57.0)	319 (92.4)	26 (7.5)	
≥4	260 (43.0)	236 (90.7)	24 (9.2)	
Household assets				0.27
<2	330 (54.5)	299 (90.6)	31 (9.3)	
≥2	275 (45.4)	256 (93.0)	19 (6.9)	
Monthly expenditures (USD)				0.03
<160	303 (50.0)	271 (89.4)	32 (10.5)	
≥160	302 (49.9)	284 (94.0)	18 (5.9)	
History of low birth weight infants				0.49
No	516 (85.3)	475 (92.0)	41 (7.9)	
Yes	89 (14.7)	80 (89.8)	9 (10.1)	
History of miscarriage				0.66
No	321 (53.0)	293 (91.2)	28 (8.7)	
Yes	284 (46.9)	262 (92.2)	22 (7.7)	
Current pregnancy				
Gestational age, trimester				0.01
First	100 (16.5)	98 (98.0)	2 (2.0)	
Second	295 (48.7)	262 (88.8)	33 (11.1)	
Third	210 (34.7)	195 (92.8)	15 (7.1)	
Pattern of prenatal visits				0.12
Irregular	140 (23.1)	124 (88.5)	16 (11.4)	
Regular	465 (76.9)	431 (92.7)	34 (7.3)	
Birth order of newborn				0.71
First	234 (38.6)	217 (92.7)	17 (7.2)	
Second to forth	354 (58.5)	323 (91.2)	31 (8.7)	
Fifth or more	17 (2.8)	15 (88.2)	2 (11.7)	
Intake of fruits/vegetables				0.004
No	258 (42.6)	227 (87.9)	31 (12.0)	
Yes	347 (57.3)	328 (94.5)	19 (5.4)	
Dietary supplements^1^				0.90
No	93 (15.4)	85 (91.3)	8 (8.6)	
Yes	512 (84.6)	470 (91.8)	42 (8.2)	
Second-hand water-pipe smoking^2^				<0.001
No	450 (74.3)	444 (98.6)	6 (1.3)	
Yes	155 (25.6)	111 (71.6)	44 (28.4)	

Values are presented as number (%).

1 Supplements included iron and multivitamin pills.

2 Exposure to water-pipe smoke at home or work.

**Table 2.** Proportion of low birth weight infants according to water-pipe smoking in pregnant women in Bandar Abbas, Iran, 2016-2017

Variable	Total n (%)	Low birth weight			Statistic (p-value)^1^
		n (%)	95% CI		
			Lower limit	Upper limit	
Overall	605 (100.0)	71 (11.7)	9.1	14.3	-
Water-pipe smoking					
No	555 (91.7)	59 (10.6)	8.0	13.2	7.9 (0.005)
Yes	50 (8.2)	12 (24.0)	12.0	35.9	
Age at starting water-pipe smoking (yr)					
Non-smoker	555 (91.7)	59 (10.6)	8.0	13.2	27.4 (<0.001)
≤-15	21 (3.4)	10 (47.6)	25.6	69.5	
>15	29 (4.8)	2 (6.9)	0.0	16.3	
Duration of water-pipe smoking (yr)					
Non-smoker	555 (91.7)	59 (10.6)	8.0	13.2	16.4 (<0.001)
<5	13 (2.1)	2 (15.3)	0.0	35.8	
5-10	13 (2.1)	1 (7.6)	0.1	22.8	
>10	24 (3.9)	9 (37.0)	17.6	57.3	

CI, confidece interval.

1 Calculated by chi-square test.

**Table 3.** The results of generalized linear models for low birth weight according to water-pipe smoking in pregnant women in Bandar Abbas, 2016-2017

Predictors	Unadjusted		Adjusted	
	RR (95% CI)	SE	RR (95% CI)	SE
Monthly expenditures (USD)				
<160	1.00 (reference)		1.00 (reference)	
≥160	1.09 (0.70, 1.69)	0.24	1.14 (0.73, 1.78)	0.26
Household size				
<4	1.00 (reference)		1.00 (reference)	
≥4	8.09 (4.22, 15.48)	2.68	8.56 (4.45, 16.44)	2.85
Household assets				
≥2	1.00 (reference)		1.00 (reference)	
<2	1.10 (0.71, 1.70)	0.24	1.16 (0.77, 1.74)	0.24
Pattern of prenatal visits				
Irregular	1.00 (reference)		1.00 (reference)	
Regular	0.76 (0.47, 1.24)	0.18	0.71 (0.45, 1.11)	0.16
Gestational age, trimester				
First	1.00 (reference)		1.00 (reference)	
Second	0.69 (0.40, 1.21)	0.19	0.64 (0.37, 1.10)	0.17
Third	0.65 (0.35, 1.19)	0.20	0.62 (0.33, 1.14)	0.19
Water-pipe smoking				
No	1.00 (reference)		1.00 (reference)	
Yes	2.25 (1.30, 3.91)	0.63	2.09 (1.18, 3.71)	0.61
Duration of water-pipe smoking (yr)	1.04 (1.02, 1.06)	0.01	1.02 (0.99, 1.05)	0.01
Second-hand water-pipe smoking				
No	1.00 (reference)		1.00 (reference)	
Yes	1.78 (1.14, 2.77)	0.40	1.21 (0.75, 1.97)	0.29

RR, relative risk; CI, confidece interval; SE, standard error.
